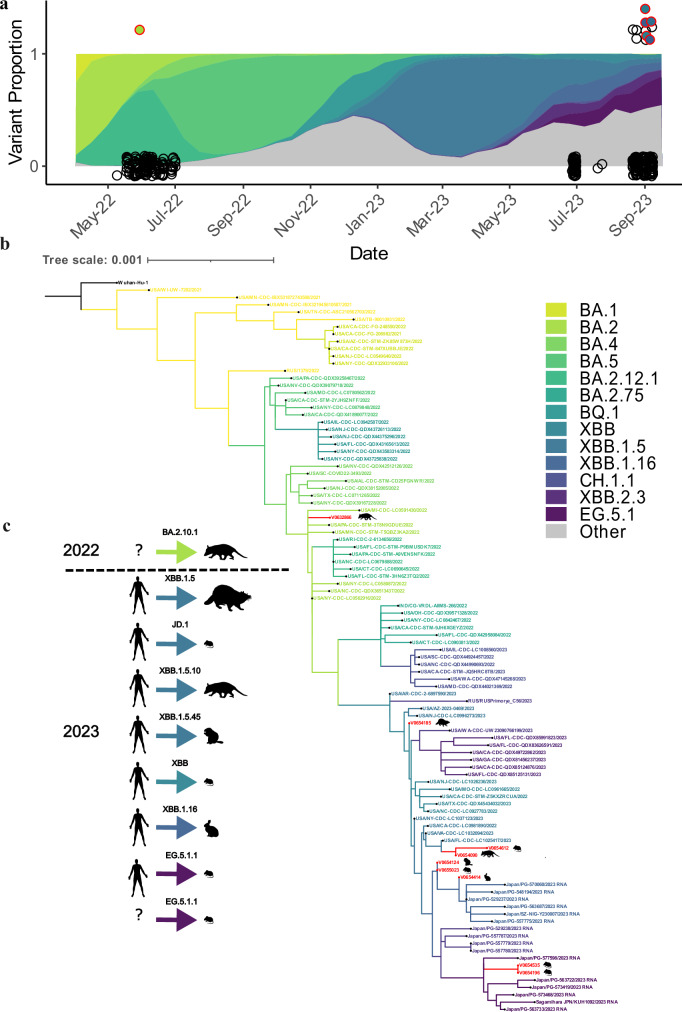# Author Correction: Widespread exposure to SARS-CoV-2 in wildlife communities

**DOI:** 10.1038/s41467-024-51220-0

**Published:** 2024-08-13

**Authors:** Amanda R. Goldberg, Kate E. Langwig, Katherine L. Brown, Jeffrey M. Marano, Pallavi Rai, Kelsie M. King, Amanda K. Sharp, Alessandro Ceci, Christopher D. Kailing, Macy J. Kailing, Russell Briggs, Matthew G. Urbano, Clinton Roby, Anne M. Brown, James Weger-Lucarelli, Carla V. Finkielstein, Joseph R. Hoyt

**Affiliations:** 1 Department of Biological Sciences, Virginia Tech, Blacksburg, VA USA; 2 Virginia Tech Carilion School of Medicine, Virginia Tech, Roanoke, VA USA; 3 Center for Emerging, Zoonotic, and Arthropod-borne Pathogens, Virginia Tech, Blacksburg, VA USA; 4 Molecular Diagnostics Laboratory, Fralin Biomedical Research Institute, Virginia Tech, Roanoke, VA USA; 5 Department of Biomedical Sciences and Pathobiology, Virginia Tech, Blacksburg, VA USA; 6 Translational Biology, Medicine, and Health Graduate Program, Virginia Tech, Roanoke, VA USA; 7 Program in Genetics, Bioinformatics, and Computational Biology, Virginia Tech, Blacksburg, VA USA; 8 Department of Biochemistry, Virginia Tech, Blacksburg, VA USA; 9 Data Services, University Libraries, Virginia Tech, Blacksburg, VA USA; 10 Virginia Tech Center for Drug Discovery, Virginia Tech, Blacksburg, VA USA; 11 Academy of Integrated Science, Virginia Tech, Blacksburg, VA USA

**Keywords:** Ecology, Urban ecology, Epidemiology, SARS-CoV-2

Correction to: *Nature Communications* 10.1038/s41467-024-49891-w, published online 29 July 2024

In this article Figure 3, panel A is missing points. The figure should have appeared as shown below. The original article has been corrected.

**Corrected**

**Figure:**
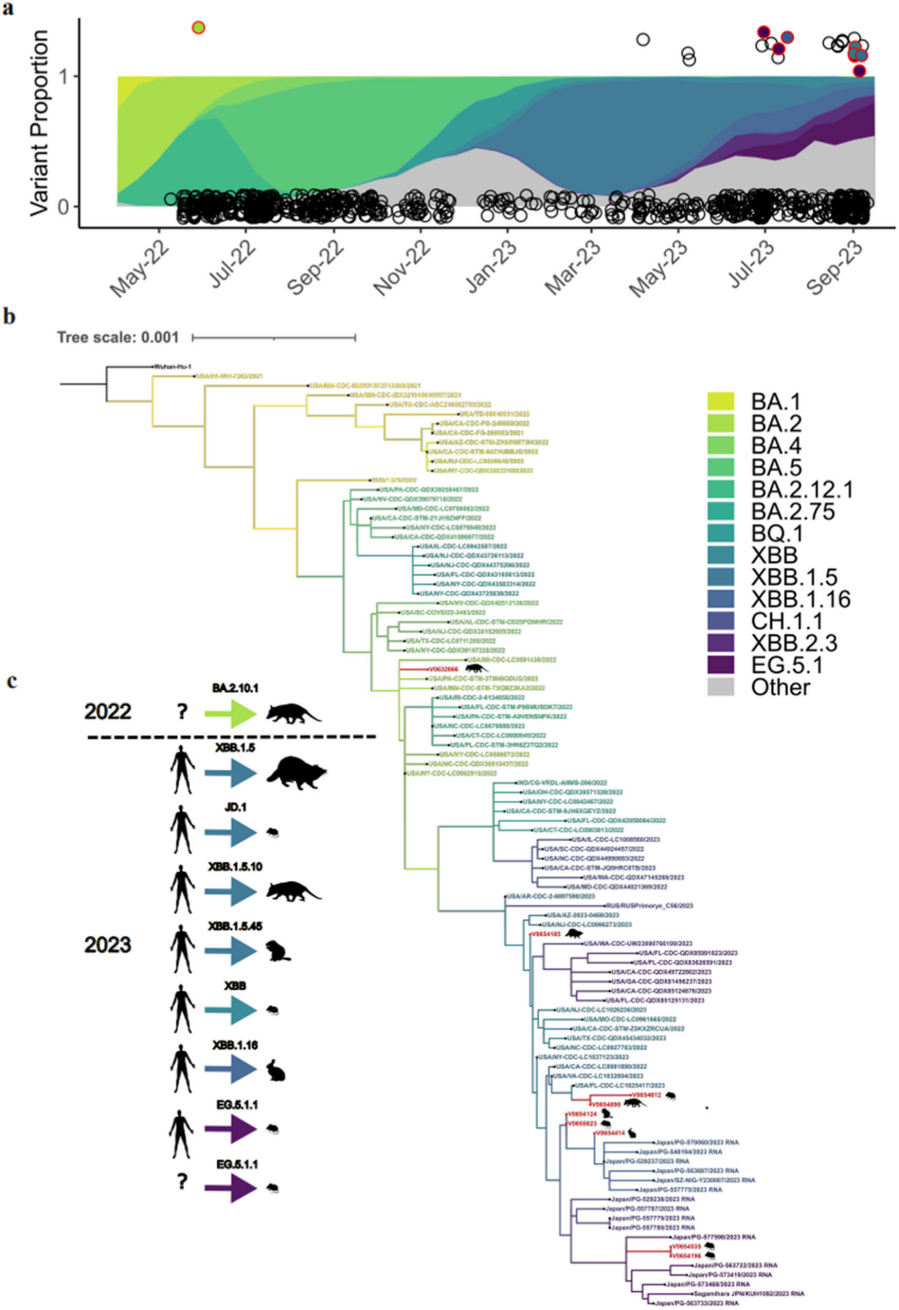

**Original**

**Figure:**