# Supplementary material for: Widespread exposure to SARS-CoV-2 in wildlife communities
Source: Nat Commun. 2024 Jul 29;15:6210. doi: 10.1038/s41467-024-49891-w (PMC11286844; doi:10.1038/s41467-024-49891-w)
Supplement: Supplementary file 1 — Supplementary Information [file 41467_2024_49891_MOESM1_ESM.pdf]

Supplementary Information for: Widespread exposure to SARS-CoV-2 in wildlife communities

Amanda R. Goldberg, Kate E. Langwig, Katherine L. Brown, Jeffrey Marano, Pallavi Rai, Kelsie M. King, Amanda K. Sharp, Alessandro Ceci, Christopher D. Kailing, Macy J. Kailing, Russell Briggs, Matthew G. Urbano, Clinton Roby, Anne M. Brown, James Weger-Lucarelli, Carla V. Finkielstein, Joseph R. Hoyt

Supplementary Information Text

Site descriptions

For all sites, traps were placed near structures (homes, sheds, and fences) as well as in and around vegetation (trees, bushes, gardens). We had two urban sites located in Blacksburg, VA (BB) and Roanoke, VA (RK). All homes were located in residential neighborhoods composed of single family or multiunit houses. Our locations in Roanoke were all on public land located in three small urban parks along the Roanoke River Greenway or on Mill Mountain Park. The small parks are located along a major bike/walking paved trail that runs along a river through the center of Roanoke. These parks were all located in mixed-use areas that included nearby houses, apartment buildings, hospitals, and businesses. Preston Forest (PF) is an intermediary site between urban and rural. Houses are spaced further apart (~5 acre lots) and hence the human population is at a lower density. Furthermore, there are no sidewalks so foot traffic is reduced. We trapped in the backyards of two homes that are mainly dominated by woody vegetation and leaf debris.

New River Trails State Park (Foster Falls; NRT) is a section of a larger State Park located along the New River. Foster Falls has a campground and is a popular area for outdoor recreation. Brush Mountain (BM) is located within a complex of private, non-profit owned lands that are open to the public for hiking and biking. The area we trapped was not yet open to the public as it was in the process of being prepared (building a parking area, grooming trails, etc). Mountain Lake Biological Station site (ML) is a research field station with a rotating group of people using the facilities, including classrooms and housing. We trapped away from the housing complex in the surrounding forest. There was a hiking trail located adjacent to our sites and a commercial lodge

about two miles down the road. However, the lands owned by the research station, where we trapped, were off-limits to public use. Pandapas Pond (PP) is located in Montgomery county and is a high use area for outdoor recreation including trail running, hiking, mountain biking, and fishing. We placed traps along a forest service road. The area is dominated by trees and leaf debris on the ground. Caldwell fields (CF) is located also in Montgomery county. The area is lightly used for general outdoor recreation including hunting.

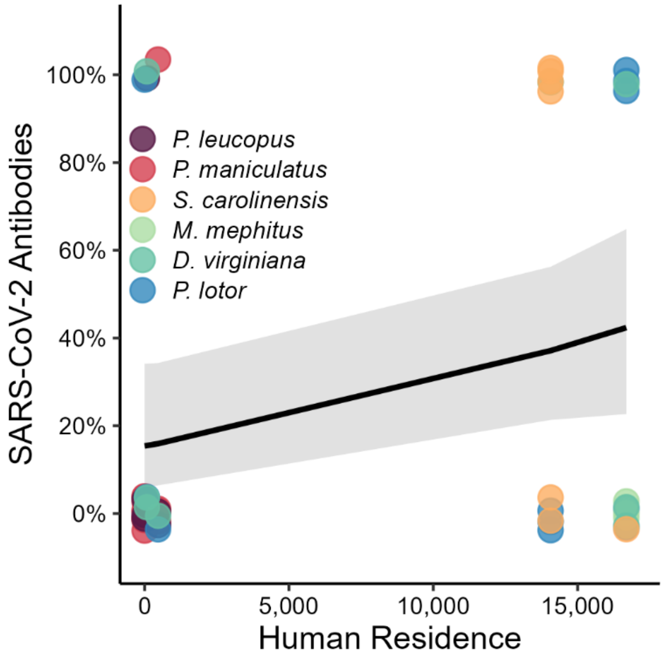

Supplementary Figure 1. Examination of SARS-CoV-2 exposure relationship between human residence (U.S. 2020 census data) and seroprevalence collected from 5 different sites in VA, USA. Black line indicates the model fit and the grey ribbons (shadowed area) represent 95% confidence intervals (intercept = -1.277, β = 0.607, P = 0.059). Color of the circle indicates the species sampled and each circle represents and individual (n=67).

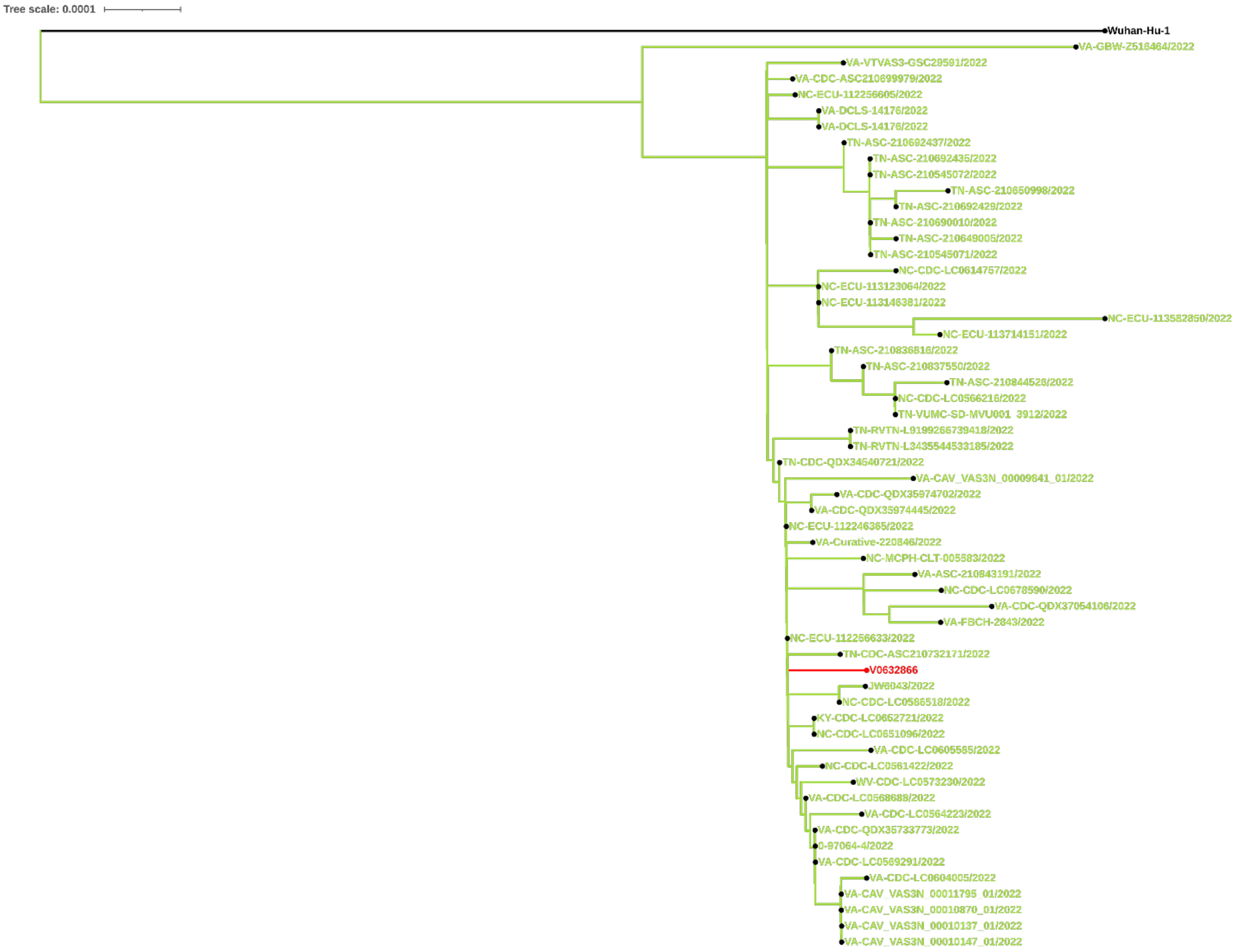

Supplementary Figure 2. Phylogenetic tree for a Virginia opossum (V0652866) assigned to the BA.2.10.1 Pango lineage. See Supplementary Table 11 for further information. Red indicates the wildlife sample.

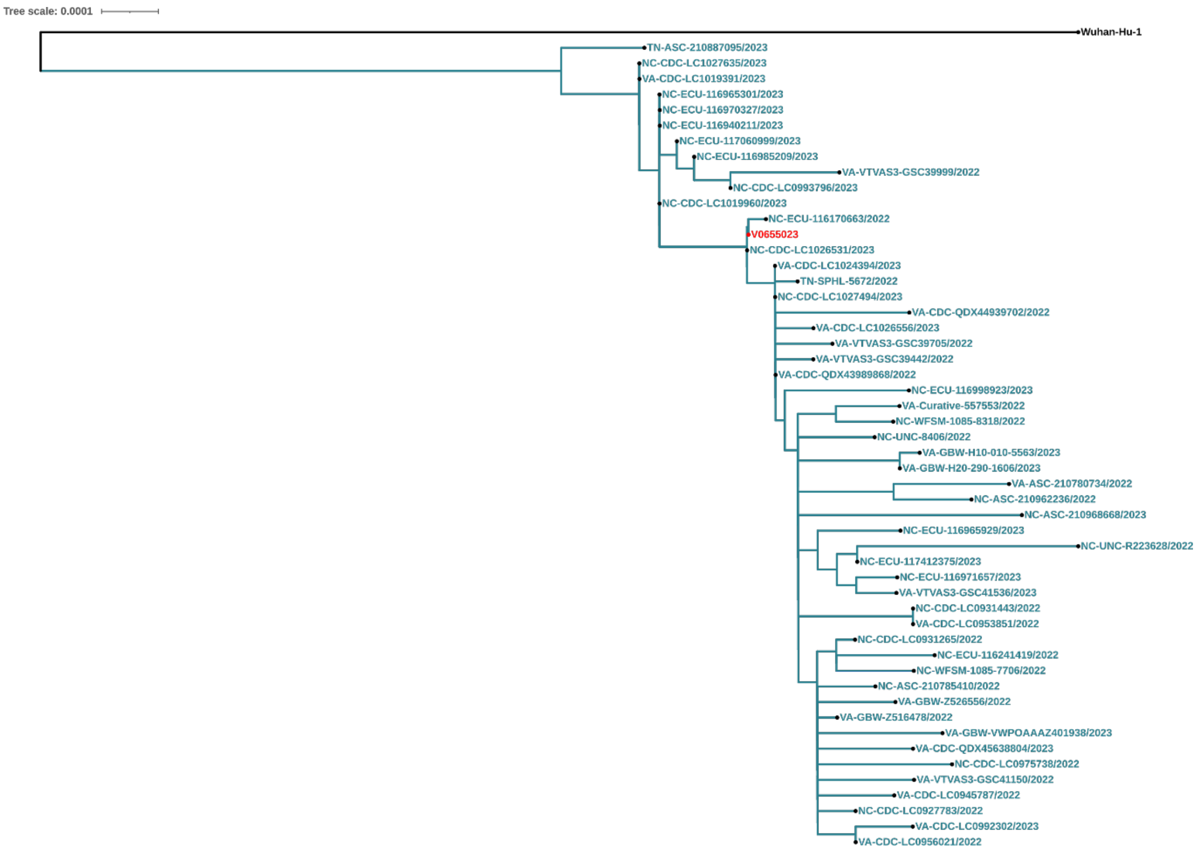

Supplementary Figure 3. Phylogenetic tree for a deer mouse (V0655023) assigned to the XBB Pango lineage. See Supplementary Table 11 for further information. Red indicates the wildlife sample.

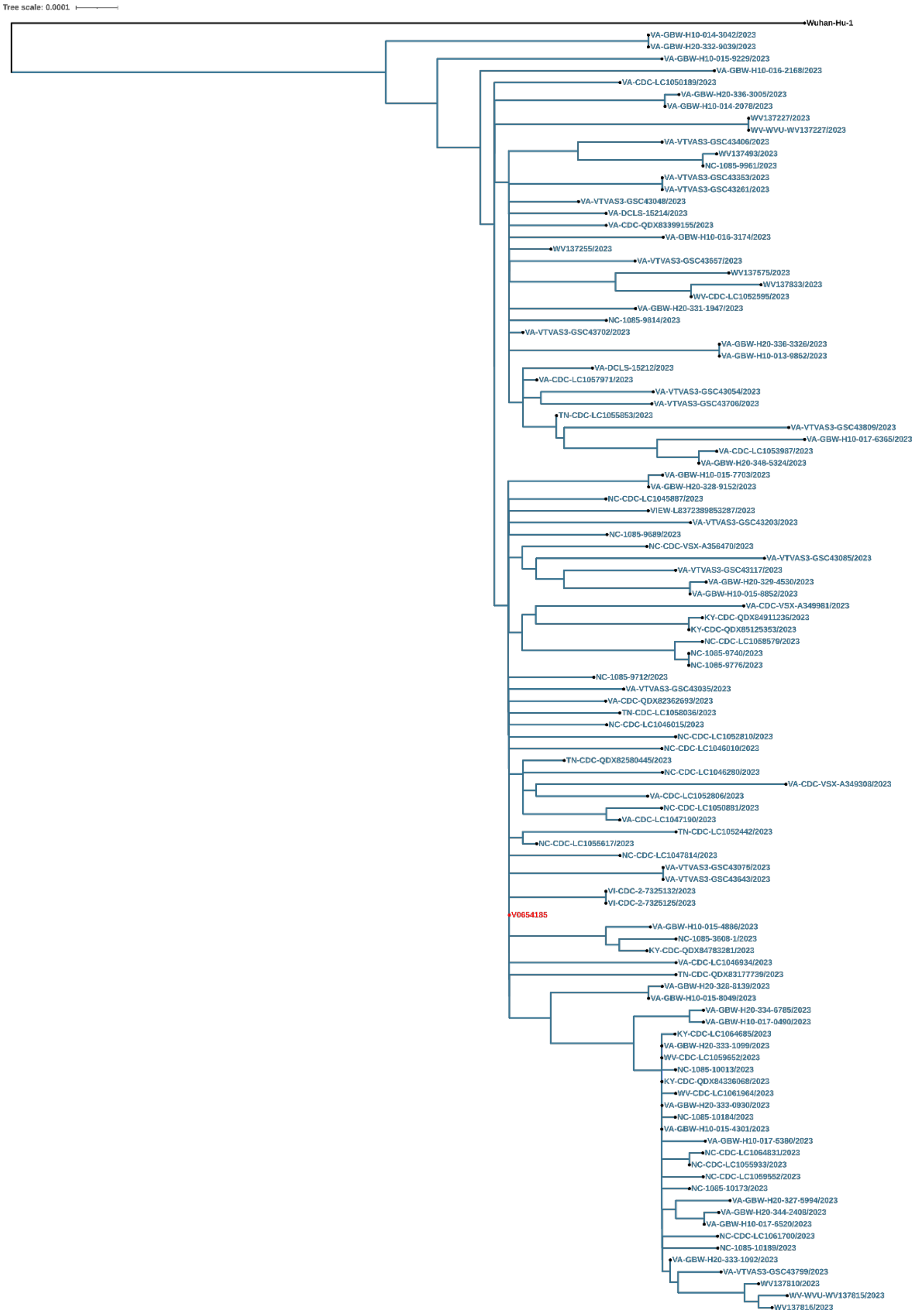

Supplementary Figure 4. Phylogenetic tree for a raccoon (V0654185) assigned to the XBB.1.5 Pango lineage. See Supplementary Table 11 for further information. Red indicates the wildlife sample.

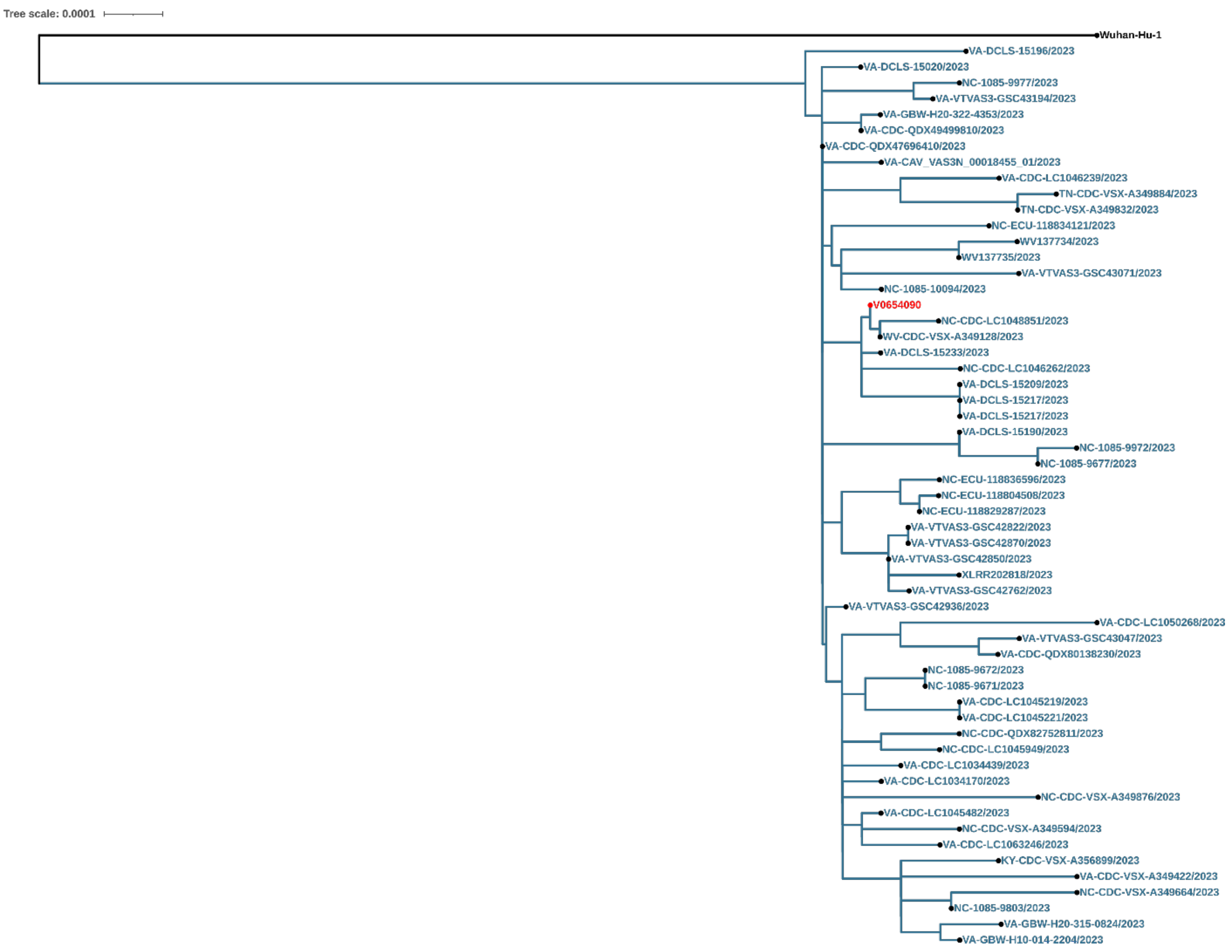

Supplementary Figure 5. Phylogenetic tree for a Virginia opossum (V0654090) assigned to the XBB.1.5.10 Pango lineage. See Supplementary Table 11 for further information. Red indicates the wildlife sample.

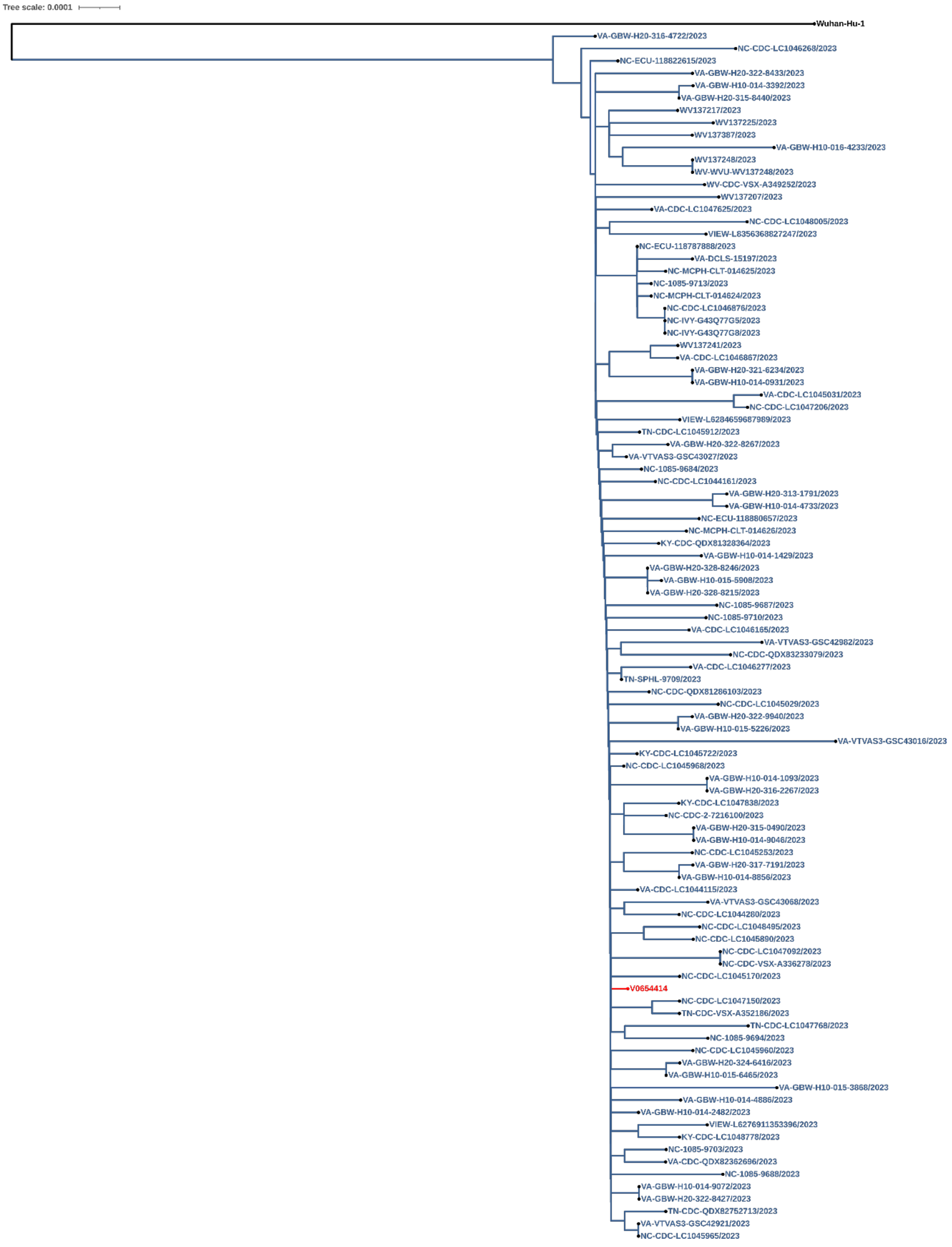

Supplementary Figure 6. Phylogenetic tree for an Eastern cottontail (V0654414) assigned to the XBB.1.16 Pango lineage. See Supplementary Table 11 for further information. Red indicates the wildlife sample.

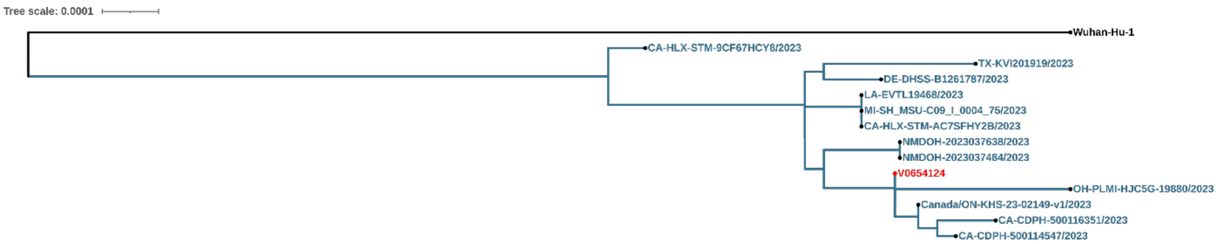

Supplementary Figure 7. Phylogenetic tree for a groundhog (V0654124) assigned to XBB.1.5.45 Pango lineage. See Supplementary Table 11 for further information. Red indicates the wildlife sample.

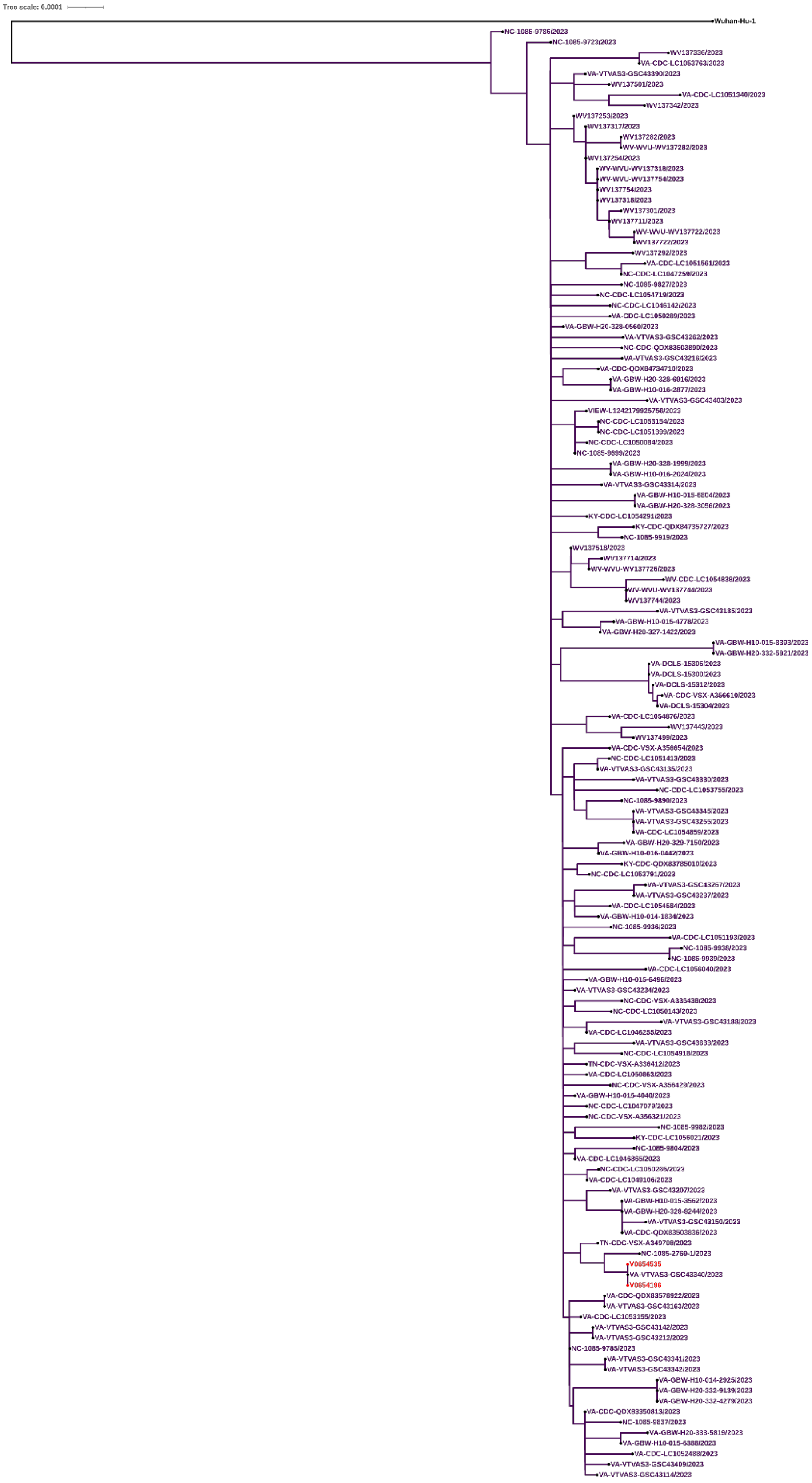

Supplementary Figure 8. Phylogenetic tree for two deer mice (V0654196 and V0653535) assigned to the EG.5.1.1 Pango lineage. See Supplementary Table 11 for further information. Red indicates the wildlife sample.

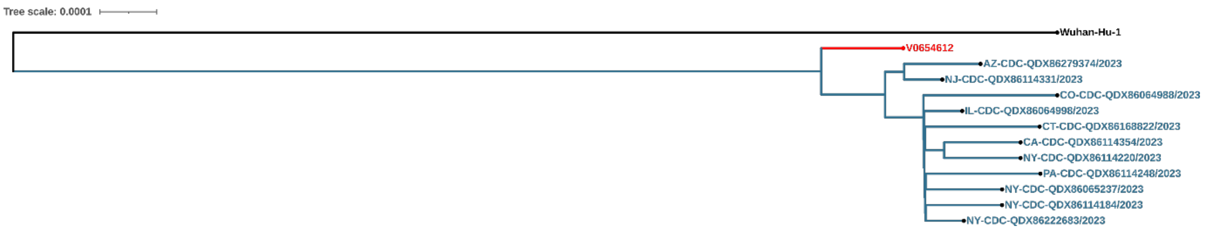

Supplementary Figure 9. Phylogenetic tree for a deer mouse (V0654612) assigned to the JD.1 Pango lineage. See Supplementary Table 11 for further information. Red indicates the wildlife sample.

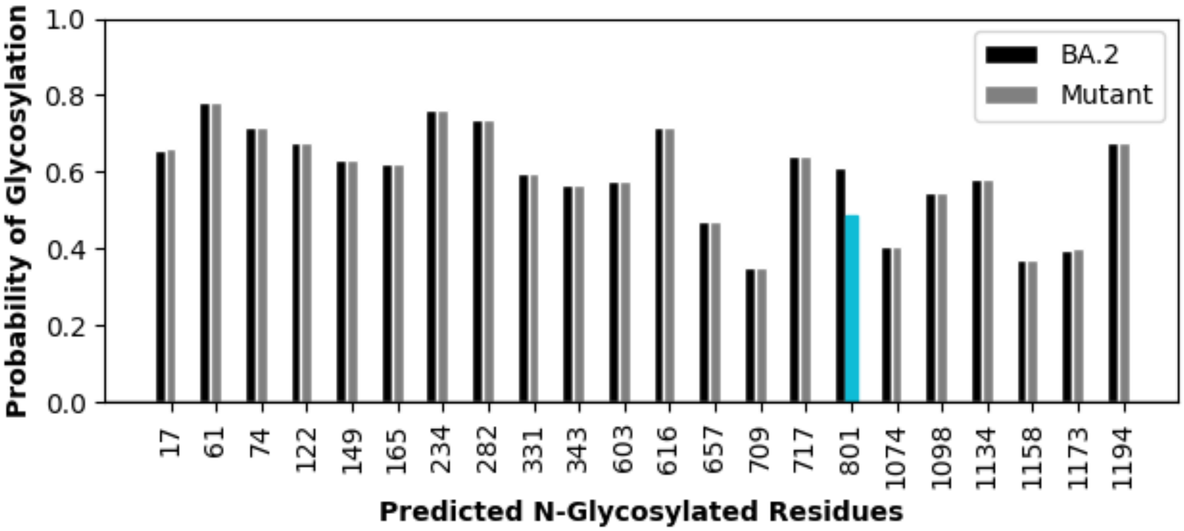

Supplementary Figure 10. Predicted N-glycosylated residues identified by the NetNGlyc 1.0 server with the probability of being glycosylated based on BA.2 or [mutant] sequence.

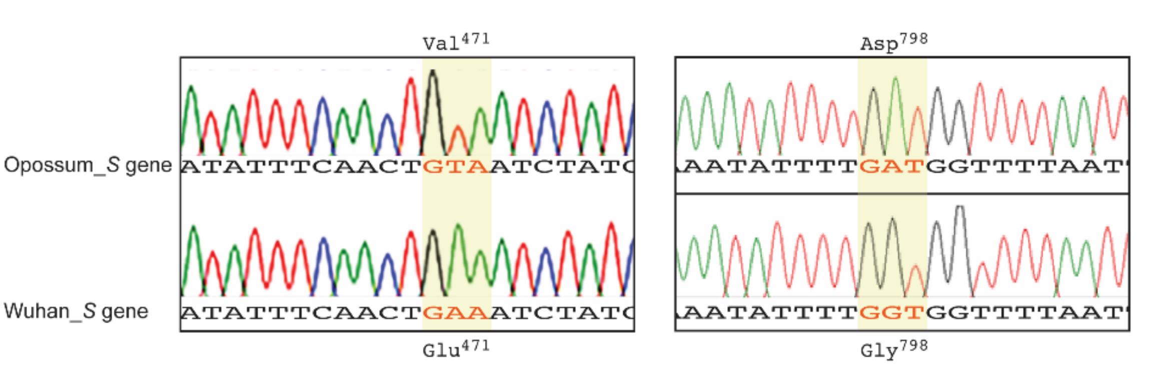

Supplementary Figure 11. Sanger sequencing peaks of the genomic regions for the positive opossum from July 2022. Flanking the Glu^{471}Val (left) and Gly^{798}Asp (right) mutations identified in the *S* gene of the opossum-infected SARS-CoV-2 sample and its corresponding sequence in the original Wuhan strain. Nucleotide mutations and matching wild-type sequences are shaded in yellow.

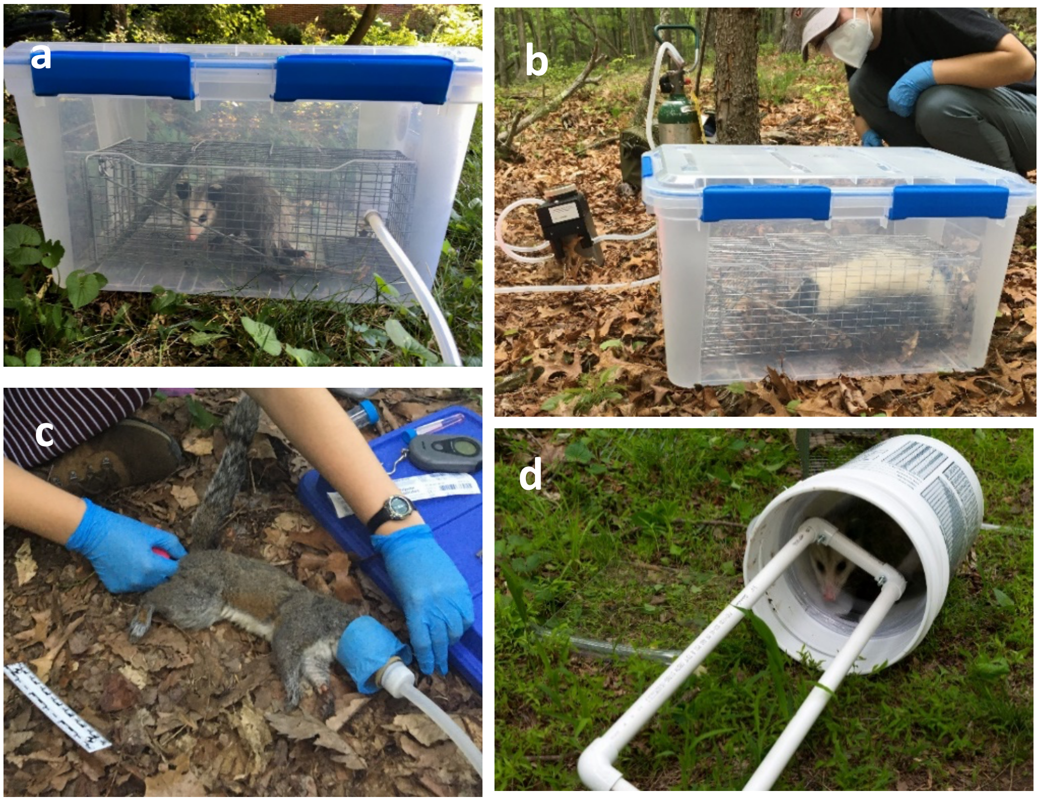

Supplementary Figure 12. Equipment modified for animal processing. A modified plastic container (a & b). We used a vaporizer with a small O2 tank to supply isoflurane into the chambers (b). We made masks from the top part of a plastic bottle (c). We used a modified bucket chamber for anesthetizing animals trapped in larger cages (d).

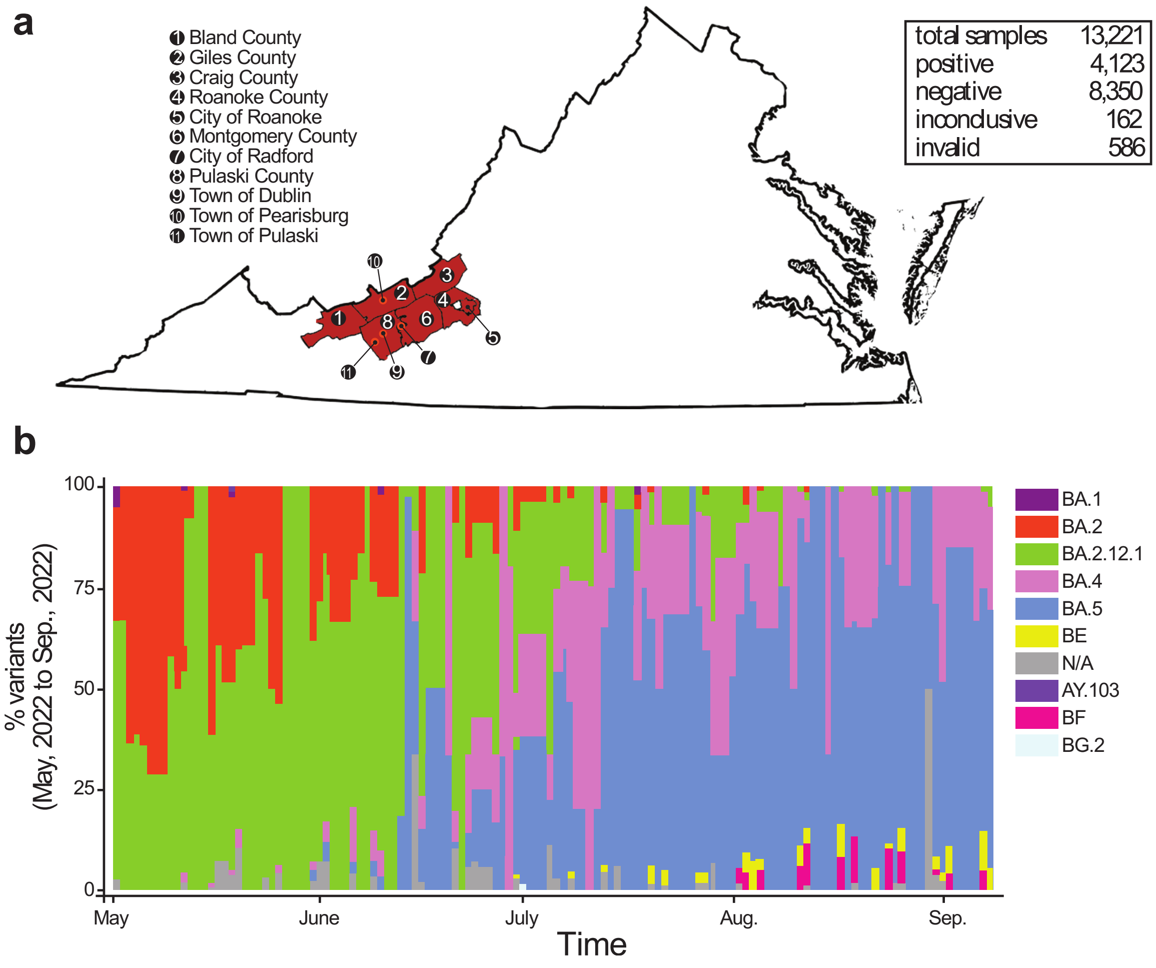

Supplementary Figure 13. Distribution of SARS-CoV-2 variants in circulation during the 2022 sampling period. (a) Map of counties where sequence data was obtained. (b) Summary of weekly distribution of SARS-CoV-2 variants circulating in human communities in Southwest Virginia between May and September, 2022 as determined by RT-qPCR, RMA, and WGS sequencing.

Supplementary Table 1. Summary of species tested including both RT-qPCR and serology (60% cut-off) results for SARS-CoV-2 after the virus had arrived in the United States.

Species	RT-qPCR (n)	RT-qPCR positive ≥2 genes[1]	RT-qPCR positive 1 gene[1]	Number of WGS obtained	Number of partial WGS obtained	Serology (n)	Sero-positive samples
Peromyscus maniculatus[2]	172	8 (4N+E+S, 1N+E, 1N+S, 2E+S)	9 (4N, 1E, 4S)	4	1	14	1
Procyon lotor[2]	84	4 (3N+E+S, 1N+S)	5 (2N, 3S)	1		11	4
Didelphis virginiana[2]	140	4 (1N+S+E, 1N+S, 1N+E, 1E+S)	16 (2N, 6E, 8S)	2		8	2
Sciurus carolinensis[2]	105		4 (1E, 3S)			7	4
Peromyscus leucopus[2]	19					6	0
Mephitis mephitis[2]	25		1 (1N)			3	2
Vulpes vulpes[2]	17		2 (1N, 1S)				
Odocoileus virginianus	20		3 (3S)				
Lynx rufus	3		2 (2S)				
Sylvilagus floridanus	118	3 (1N+E+S, 1N+S, 1E+S)	9 (7N, 1E 1S)	1			
Marmota monax[2]	31	3 (2N+E, 1E+S)	2 (2N)	1			
Tamias striatus[2]	12						
Lasiurus borealis[2]	12	1 (1N+S)	1 (1N)	0	2		
Eptesicus fuscus[2]	7						
Ursus americanus	7		1 (1N)				
Sciurus niger	3						
Blarina brevicada[2]	3						
Rattus rattus	2						
Urocyon cinereoargenteus	2						
Castor canadensis	2		1 (1S)				
Peromyscus sp.	2						
Mustela vison	1						
Microtus pennsylvanicus	1						
Mus musculus	1						
Total	789	23	53	9	3	49	24

[1]Letter denotes SARS-CoV-2 genes(s) that amplified.
[2]Species sampled at one of the 8 study sites

Supplementary Table 2. Comparative analysis of SARS-CoV-2 sequences from wild animals isolated in southwest Virginia. Consensus sequences for SARS-CoV-2 isolated from wild animal specimen were analyzed for sequence coverage and percent identity compared to the SARS-CoV-2 reference genome (NC_045512), Pango lineage, closely related SARS-CoV-2 sequence isolated from a human, and unique amino acid substitutions, which are not found in their closely related sequences. See Table S5 for unresolved sequences.

Sample ID (Accession number)	Animal species	Location	Date of isolation	Sequence coverage (Percent Identity)	Pango	Closely related sequence, state & collection date	Unique amino acid substitutions
V0632866 (OR866905)	*Didelphis virginiana*	Blacksburg, Montgomery Co, VA	05/29/2022	96.8% (99.8%)	BA.2.10.1	OM999909 TN 03/06/2022	ORF1a:T2495I S:E471V
V0654090 (OR878666)	*Didelphis virginiana*	Star Tannery, Frederick Co, VA	07/10/2023	31.8% (99.0%)	XBB.1.5.10	OR454205 VA 7/18/2023	none
V0654124 (OR866349)	*Marmota monax*	Front Royal, Warren Co, VA	06/30/2023	73.5% (99.8%)	XBB.1.5.45	EPI_ISL_17744393 OH 06/02/2023*	S:H146Q
V0654196 (OR866382)	*Peromyscus maniculatus*	Blacksburg, Montgomery Co, VA	09/02/2023	99.1% (99.5%)	EG.5.1.1	EPI_ISL_18287981 VA 08/29/2023	none
V0654535 (OR866443)	*Peromyscus maniculatus*	Blacksburg, Montgomery Co, VA	09/02/2023	88.6% (99.4%)	EG.5.1.1	EPI_ISL_18287981 VA 08/29/2023	none
V0654612 (OR878668)	*Peromyscus maniculatus*	Blacksburg, Montgomery Co, VA	09/02/2023	24.3% (99.1%)	JD.1	OR708231 NJ 10/06/23	none
V0655023 (OR866910)	*Peromyscus maniculatus*	Blacksburg, Montgomery Co, VA	09/06/2023	59.7% (99.8%)	XBB	EPI_ISL_16384582 NC 11/26/22	none
V0654185 (OR878667)	*Procyon lotor*	Max Meadows, Wythe Co, VA	09/06/2023	25.0% (99.6%)	XBB.1.5	EPI_ISL_18124823 VA 08/13/2023	none
V0654414 (OR866437)	*Sylvilagus floridanus*	Stafford, Stafford Co, VA	07/17/2023	82.1% (99.8%)	XBB.1.16	OR252035 NC 06/22/23	none

Supplementary Table 3. Summary of SARS-CoV-2 amplicon sequences obtained from wild animals. Sequences were analyzed for their length in bases and their position and percent identity relative to the reference genome (NC_045512.2).

Sample ID (Accession number)	Animal Species	Location	Date	Sequence length	SARS-CoV-2 sequence position	Percent identity (mutation count)
V0654648 (OR871756 OR872533)	*Lasiurus borealis*	Blacksburg, Montgomery Co, VA	8/21/2023	272<br>267	24508..24779<br>28516..28782	100% (0)<br>100% (0)
V0655027 (OR871072)	*Lasiurus borealis*	Blacksburg, Montgomery Co, VA	8/21/2023	249	24812..25060	99% (2)
V0654636 (OR871750 OR872518 OR871751)	*Peromyscus maniculatus*	Blacksburg, Montgomery Co, VA	09/02/2023	377<br>247<br>317	23570..23946<br>28196..28451<br>28464..28780	99% (3)<br>95% (12)<br>100% (0)

Supplementary Table 4. Number of seropositive samples under 4 different percent neutralization cutoff values from the 49 samples collected in summer 2022 in Virginia, U.S.A from 6 species. The four different percent neutralization cutoff values we evaluated include: 40% (Pos 40), 50% (Pos 50), 60% (Pos 60; the value we used for Fig 2c), and 70% (Pos 70). Additionally, we include the seropositivity values for each species under the 4 different cutoff values.

Species	Pos 40	Pos 50	Pos 60	Pos 70	N	Percent Pos 40	Percent Pos 50	Percent Pos 60	Percent Pos 70
Peromyscus leucopus	2	1	1	1	6	33.33%	16.67%	16.67%	16.67%
Peromyscus maniculatus	6	4	1	0	14	42.86%	28.57%	7.14%	0.00%
Sciurus carolinensis	6	5	4	2	7	85.71%	71.43%	57.14%	28.57%
Mephitus mephitus	2	2	0	0	3	66.67%	66.67%	0.00%	0.00%
Didelphis virginiana	6	5	3	1	8	75.00%	62.50%	37.50%	12.50%
Procyon lotor	9	7	4	3	11	81.82%	63.64%	36.36%	27.27%
Total	31	24	13	7	49	63.27%	48.98%	26.53%	14.29%

Supplementary Table 5. Generalized linear mixed model results evaluating the relationship between urbanization and seroprevalence of mammals with species as a random effect. We evaluated this relationship using 5 different % neutralization cutoff values. All reported P values are two-tailed and the model consisted of a single predictor variable and multiple comparisons were not made. See Fig. 2 for predicted relationship from the 60% cutoff model.

% Neutralization	Intercept			Imperviousness		
cutoff	β	SE	p	β	SE	p
40%	-0.054	0.372	0.884	0.053	0.024	0.026
50%	-0.608	0.380	0.109	0.042	0.019	0.024
60%	-1.655	0.477	0.001	0.038	0.018	0.032
65%	-1.892	0.514	0.000	0.044	0.019	0.017
70%	-1.885	0.561	0.001	0.005	0.179	0.858

Supplementary Table 6. Summary of serology and RT-qPCR results from samples collected in Virginia and Washington D.C. in 2022 and 2023.

Species	Serology (n)	Sero-positive (Y/N)	RT-qPCR (n)	RT-qPCR positive (Y/N)	WGS (Y/N)	Partial Sequence (Y/N)
Peromyscus maniculatus	14	Y	172	Y	Y	Y
Procyon lotor	11	Y	84	Y	Y	N
Didelphis virginiana	8	Y	140	Y	Y	N
Sciurus carolinensis	7	Y	105	N	N	N
Peromyscus leucopus	6	N	19	N	N	N
Mephitis mephitis	3	Y	25	N	N	N
Vulpes vulpes			17	N	N	N
Odocoileus virginianus			20	N	N	N
Lynx rufus			3	N	N	N
Sylvilagus floridanus			118	Y	Y	N
Marmota monax			31	Y	Y	N
Tamias striatus			12	N	N	N
Lasiurus borealis			12	Y	N	Y
Eptesicus fuscus			7	N	N	N
Ursus americanus			7	N	N	N
Sciurus niger			3	N	N	N
Blarina brevicada			3	N	N	N
Rattus rattus			2	N	N	N
Urocyon cinereoargenteus			2	N	N	N
Castor canadensis			2	N	N	N
Peromyscus sp.			2	N	N	N
Mustela vison			1	N	N	N
Microtus pennsylvanicus			1	N	N	N
Mus musculus			1	N	N	N

Supplementary Table 7. Comparison of results from this study, experimental infection studies and predicted susceptibility based on modeling of the ACE2 receptor. RT-qPCR and seroprevalence data from our study are combined with data from previously published research on whether species were capable of being infected in the lab and their predicted susceptibility based on modeling of the ACE2 receptor. For species that do not have an exact match we included closely related species, which are indicated after the semicolon. * Indicates instances where a species has not been evaluated but information for a closely related species is available.

Common Name	RT-qPCR Prevalence[1]	Seroprevalence[1]	Experimental Infection	ACE2 modeling
Deer mouse	5%	7%	Seroconverted, live virus isolation[3-5]	High[5-8], medium[9]
Striped Skunk	0%	0%	Seroconverted[3,10], live virus isolation[3,10]	Very low (Western spotted skunk)[9]
Raccoon	5%	36%	Seroconverted, no virus isolated[10]	Low[6,8]
White-footed mouse	0%	17%		High[5,7], low[8]
Grey squirrel	0%	57%	*No seroconversion or virus isolation (Fox squirrel)[3]	*High (Red squirrel)[11]
Virginia opossum	3%	38%		*High (Gray short-tailed opossum)[7]
Eastern red bat	8%	-	*Seroconverted, live virus isolation (Egyptian fruit bat[12]; Mexican free-tailed bat[13]. No seroconversion or virus isolation (Big brown bats)[14]	*Low-high depending on bat species[7,9,15]
Groundhog	7%	-		*Low (Alpine marmot, Yellow-bellied marmot)[16], medium (Alpine marmot)[7,9,16,17]
White-tailed deer	0%	-	Seroconverted, live virus isolation[18]	High[6,9], low[7]
Eastern cottontail	3%	-	*No seroconversion or virus isolation (European cottontail)[3]	*Low (European rabbit)[6], Medium (European rabbit)[9,16]
Eastern chipmunk	0%	-		*Low (Thirteen-lined ground squirrel)[9,16], Medium (Daurian ground squirrel[9], thirteen-lined ground squirrel[9])
Red fox	0%	-	Seroconverted, live virus isolation[19]	Low[9], medium[7]

[1]Prevalence calculated from data collected in this study. All samples were collected from wildlife in Virginia from May 2022-Sept 2023. RT-qPCR results were considered positive if they had at least two of the three genes tested as positive (N, E, and S with a Ct<40)

Supplementary Table 8. Estimates of monthly human presence at the 5 sites we trapped and collected serological data.

Site	County	Monthly Use Estimates	Human presence Group
Mountain Lake Biological Station	Giles	350	Low
Brush Mountain	Montgomery	10	Low
Blacksburg	Montgomery	84,188	High
Roanoke Parks	Roanoke	79,324	High
New River Trails State Park	Wythe	6,949	High

Supplementary Table 9. Average and maximum lifespan of wild animals based on published literature.

Species	Average lifespan in the wild (years); max is in parenthesis
Procyon lotor	5[a] (20[b])
Didelphis virginiana	1.5-2[a]
Mephitis mephitis	<1[a] (6[b])
Sciurus carolinensis	(12.5[b])
Peromyscus maniculatus	<1[a]
Peromyscus leucopus	1[a]
Blarina brevicauda	(2.5[b])
Marmota monax	4-6[a]
Lasiurus borealis	
Sylvilagus floridanus	<3[a] (5[b])
Tamias striatus	<2[a] (3[b])
Vulpes vulpes	3[a] (7[b])

[a] Myers, P., R. Espinosa, C. S. Parr, T. Jones, G. S. Hammond, and T. A. Dewey. 2024. The Animal Diversity Web (online). Accessed at https://animaldiversity.org

[b]Carey, J. R., & Judge, D. S. 2002. Longevity records: Life spans of mammals, birds, amphibians, reptiles, and fish. Monographs on population aging, (8).

Supplementary Table 10. Buffer width set for use to estimate imperviousness and population density. Buffer width differed by species to represent the space used by each individual trapped.

Species	Buffer width (m)	Source
Peromyscus spp.	50	Wolff, J. O. 1985. The effects of density, food, and interspecific interference on home range size in *Peromyscus leucopus* and *Peromyscus maniculatus*. Canadian Journal of Zoology 63:2657-2662.
Sciurus carolinensis	225	Koprowski, J. L., K. E. Munroe, and A. J. Edelman. 2016. Gray not grey: Ecology of Sciurus carolinensis in their native range in North America. The Grey Squirrel: ecology & management of an invasive species in Europe. Woodbridge, Suffolk UK: European Squirrel Initiative, 1-18.
Didelphis virginiana	500	Gallo, T., et al. 2022. Mammals adjust diel activity across gradients of urbanization. Elife 11:e74756.
Mephitis mephitis	1000	Gallo, T., et al. 2022. Mammals adjust diel activity across gradients of urbanization. Elife 11:e74756.
Procyon lotor	1000	Gallo, T., et al. 2022. Mammals adjust diel activity across gradients of urbanization. Elife 11:e74756.

Supplementary Table 11. Summary of sequences used to assemble phylogenetic trees for comparing the SARS-CoV-2 sequences isolated from wild animals to those isolated from humans in our geographical region. All human sequences were sourced from NCBI and GISAID.

Pango lineage	Geographical region	Collection date ranges	Non-duplicate sequences
BA.2.10.1 (21L)	KY, NC, TN, VA, WV	01/01/2022 - 10/19/2023	58
XBB.1.5.10 (23A)	KY, NC, TN, VA, WV	06/01/2023 - 10/19/2023	59
XBB.1.5.45 (23A)	North America	01/01/2022 - 10/21/2023	14
EG.5.1.1 (23F)	KY, NC, TN, VA, WV	07/01/2023 - 09/01/2023	139
JD.1	North America	01/01/2022 - 10/21/2023	11
XBB (22F)*	KY, NC, TN, VA, WV	09/01/2022 - 10/19/2023	53
XBB.1.5 (23A)	KY, NC, TN, VA, WV	07/01/2023 - 10/19/2023	109
XBB.1.16 (23B)	KY, NC, TN, VA, WV	06/01/2023 - 08/01/2023	99

*Only sequences from GISAID were included

SI References

1 National Ecological Observatory Network. NEON Biorepository Mammal Collection (Blood Samples). *Occurrence dataset https://doi.org/10.15468/vqy6fj accessed via the NEON Biorepository Data Portal, https://biorepo.neonscience.org/ on 2023-05-08.* (2023).

2 Gomez, A. *et al.* Land use and West Nile Virus seroprevalence in wild mammals. *Emerg Infect Dis* **14**, 962-965 (2008). https://doi.org:10.3201/eid1406.070352

3 Bosco-Lauth, A. M. *et al.* Peridomestic mammal susceptibility to severe acute respiratory syndrome coronavirus 2 infection. *Emerg Infect Dis* **27**, 2073 (2021). https://doi.org:10.3201/eid2708.210180

4 Fagre, A. *et al.* SARS-CoV-2 infection, neuropathogenesis and transmission among deer mice: Implications for spillback to New World rodents. *PLoS Pathog.* **17**, 23 (2021). https://doi.org:10.1371/journal.ppat.1009585

5 Griffin, B. D. *et al.* SARS-CoV-2 infection and transmission in the North American deer mouse. *Nature Communications* **12**, 10 (2021). https://doi.org:10.1038/s41467-021-23848-9

6 Fischhoff, I. R., Castellanos, A. A., Rodrigues, J. P., Varsani, A. & Han, B. A. Predicting the zoonotic capacity of mammals to transmit SARS-CoV-2. *Proceedings of the Royal Society B* **288**, 20211651 (2021). https://doi.org:10.1098/rspb.2021.1651

7 Samanta, A., Alam, S. S. M., Ali, S. & Hoque, M. Evaluating the transmission feasibility of SARS-CoV-2 Omicron (B.1.1.529) variant to 143 mammalian hosts: insights from S protein RBD and host ACE2 interaction studies. *Functional & Integrative Genomics* **23** (2023). https://doi.org:10.1007/s10142-023-00962-z

8 Wu, L. L. *et al.* Broad host range of SARS-CoV-2 and the molecular basis for SARS-CoV-2 binding to cat ACE2. *Cell Discov.* **6**, 12 (2020). https://doi.org:10.1038/s41421-020-00210-9

9 Damas, J. *et al.* Broad host range of SARS-CoV-2 predicted by comparative and structural analysis of ACE2 in vertebrates. *P Natl Acad Sci USA* **117**, 22311-22322 (2020). https://doi.org:10.1073/pnas.2010146117

10 Francisco, R. *et al.* Experimental Susceptibility of North American Raccoons (*Procyon lotor*) and Striped Skunks (*Mephitis mephitis*) to SARS-CoV-2. *Front Vet Sci* **8**, 10 (2022). https://doi.org:10.3389/fvets.2021.715307

11 Pach, S. *et al.* ACE2-Variants Indicate Potential SARS-CoV-2-Susceptibility in Animals: A Molecular Dynamics Study. *Molecular Informatics* **40** (2021). https://doi.org:10.1002/minf.202100031

12 Schlottau, K. *et al.* SARS-CoV-2 in fruit bats, ferrets, pigs, and chickens: an experimental transmission study. *Lancet Microbe* **1**, E218-E225 (2020). https://doi.org:10.1016/s2666-5247(20)30089-6

13 Hall, J. S. *et al.* Experimental Infection of Mexican Free-Tailed Bats (*Tadarida brasiliensis*) with SARS-CoV-2. *Msphere* **8** (2023). https://doi.org:10.1128/msphere.00263-22

14 Hall, J. S. *et al.* Experimental challenge of a North American bat species, big brown bat (*Eptesicus fuscus*), with SARS-CoV-2. *Transboundary and Emerging Diseases* **68**, 3443-3452 (2021). https://doi.org:10.1111/tbed.13949

15 Yan, H. *et al.* ACE2 receptor usage reveals variation in susceptibility to SARS-CoV and SARS-CoV-2 infection among bat species. *Nature Ecology & Evolution* **5**, 600-608 (2021). https://doi.org:10.1038/s41559-021-01407-1

16 Kumar, A. *et al.* Predicting susceptibility for SARS-CoV-2 infection in domestic and wildlife animals using ACE2 protein sequence homology. *Zoo Biology* **40**, 79-85 (2021). https://doi.org:10.1002/zoo.21576

17 Huang, X. Q., Zhang, C. X., Pearce, R., Omenn, G. S. & Zhang, Y. Identifying the Zoonotic Origin of SARS-CoV-2 by Modeling the Binding Affinity between the Spike Receptor-Binding Domain and Host ACE2. *Journal of Proteome Research* **19**, 4844-4856 (2020). https://doi.org:10.1021/acs.jproteome.0c00717

18 Palmer, M. V. *et al.* Susceptibility of White-Tailed Deer (*Odocoileus virginianus*) to SARS-CoV-2. *Journal of Virology* **95** (2021). https://doi.org:10.1128/jvi.00083-21

19 Porter, S. M., Hartwig, A. E., Bielefeldt-Ohmann, H., Bosco-Lauth, A. M. & Root, J. J. Susceptibility of Wild Canids to SARS-CoV-2. *Emerg Infect Dis* **28**, 1852-1855 (2022). https://doi.org:10.3201/eid2809.220223
