# Supplementary material for: Widespread exposure to SARS-CoV-2 in wildlife communities
Source: Nat Commun. 2024 Jul 29;15:6210. doi: 10.1038/s41467-024-49891-w (PMC11286844; doi:10.1038/s41467-024-49891-w)
Supplement: Supplementary file 3 — Description of Additional Supplementary Files [file 41467_2024_49891_MOESM3_ESM.pdf]

Description of Additional Supplementary Files

File Name: Supplementary Data 1
Description: Average RT-qPCR values for all samples in Virginia and Washington DC from June 2022 through September 2023. Samples were collected either in the field (Study) or by a Virginia wildlife rehabilitation center (Blue Ridge Wildlife Center (BRWC), The Wildlife Center of Virginia (TWCV), or Southwest Virginia Wildlife Center of Roanoke (SWVAWC)). See attached excel file.

File Name: Supplementary Data 2
Description: Serology results from the 49 samples collected after SARS-CoV-2 arrival (post) and 67 samples prior to SARS-CoV-2 arrival (pre) samples collected. All 'pre' *Peromyscus* species were provided by NEON[1], the Eastwood lab at Virginia Tech, or the Kilpatrick lab at UC Santa Cruz[2]. All counties are located in Virginia unless otherwise noted. See attached excel file.

File Name: Supplementary Data 3
Description: Housekeeping primer sequences for RT-qPCR testing in 22 wildlife species for presence of SARS-CoV-2. See attached excel file.

File Name: Supplementary Data 4
Description: Cross-reference tables for the eight phylogenetic trees (Fig 3 & Supplementary Figures 2-9). See attached.
