# Supplementary material for: Widespread exposure to SARS-CoV-2 in wildlife communities
Source: Nat Commun. 2024 Jul 29;15:6210. doi: 10.1038/s41467-024-49891-w (PMC11286844; doi:10.1038/s41467-024-49891-w)
Supplement: Supplementary file 8 — Reporting Summary [file 41467_2024_49891_MOESM8_ESM.pdf]

nature portfolio

Corresponding author(s): Joseph Hoyt

Last updated by author(s): Nov 27, 2023

Reporting Summary

Nature Portfolio wishes to improve the reproducibility of the work that we publish. This form provides structure for consistency and transparency in reporting. For further information on Nature Portfolio policies, see our Editorial Policies and the Editorial Policy Checklist.

Statistics

For all statistical analyses, confirm that the following items are present in the figure legend, table legend, main text, or Methods section.

n/a	Confirmed	
☐	☒	The exact sample size (*n*) for each experimental group/condition, given as a discrete number and unit of measurement
☐	☒	A statement on whether measurements were taken from distinct samples or whether the same sample was measured repeatedly
☐	☒	The statistical test(s) used AND whether they are one- or two-sided<br>*Only common tests should be described solely by name; describe more complex techniques in the Methods section.*
☐	☒	A description of all covariates tested
☒	☐	A description of any assumptions or corrections, such as tests of normality and adjustment for multiple comparisons
☐	☒	A full description of the statistical parameters including central tendency (e.g. means) or other basic estimates (e.g. regression coefficient) AND variation (e.g. standard deviation) or associated estimates of uncertainty (e.g. confidence intervals)
☐	☒	For null hypothesis testing, the test statistic (e.g. *F*, *t*, *r*) with confidence intervals, effect sizes, degrees of freedom and *P* value noted<br>*Give P values as exact values whenever suitable.*
☒	☐	For Bayesian analysis, information on the choice of priors and Markov chain Monte Carlo settings
☒	☐	For hierarchical and complex designs, identification of the appropriate level for tests and full reporting of outcomes
☒	☐	Estimates of effect sizes (e.g. Cohen's *d*, Pearson's *r*), indicating how they were calculated

Our web collection on statistics for biologists contains articles on many of the points above.

Software and code

Policy information about availability of computer code

Data collection	NA
Data analysis	All data was analyzed using the following programs: Program R v 4.1.1, ARCGIS v 10.6, ONT's Guppy software, ONT's EPI2ME Labs software package,DECIPHER software package,NextClade, PyMOL, NetNGlyc 1.0 server.

For manuscripts utilizing custom algorithms or software that are central to the research but not yet described in published literature, software must be made available to editors and reviewers. We strongly encourage code deposition in a community repository (e.g. GitHub). See the Nature Portfolio guidelines for submitting code & software for further information.

Data

Policy information about availability of data

All manuscripts must include a data availability statement. This statement should provide the following information, where applicable:

- Accession codes, unique identifiers, or web links for publicly available datasets
- A description of any restrictions on data availability
- For clinical datasets or third party data, please ensure that the statement adheres to our policy

All study data are included in the article and supporting information. Sequence data from SARS-CoV-2 viruses from Virginia opossum sequenced in this study are available in the GISAID database (https://www.gisaid.org/) and NCBI (https://ncbi.nlm.nih.gov) SARS-CoV-2 sequence repositories. Accession numbers can be found in Supplemental Tables 2 and 3 and as follows OR866905 [https://www.ncbi.nlm.nih.gov/nuccore/OR866905], OR878666[https://www.ncbi.nlm.nih.gov/nuccore/

OR878666], OR866349[https://www.ncbi.nlm.nih.gov/nuccore/ OR866349], OR866382[https://www.ncbi.nlm.nih.gov/nuccore/ OR866382], OR866443[https://www.ncbi.nlm.nih.gov/nuccore/ OR866443], OR878668[https://www.ncbi.nlm.nih.gov/nuccore/ OR878668], OR866910[https://www.ncbi.nlm.nih.gov/nuccore/ OR866910], OR878667[https://www.ncbi.nlm.nih.gov/nuccore/ OR878667], OR866437[https://www.ncbi.nlm.nih.gov/nuccore/ OR866437], OR871756[https://www.ncbi.nlm.nih.gov/nuccore/OR871756], OR872533[https://www.ncbi.nlm.nih.gov/nuccore/ OR872533], OR871072[https://www.ncbi.nlm.nih.gov/nuccore/ OR871072], OR871750[https://www.ncbi.nlm.nih.gov/nuccore/ OR871750], OR871751[https://www.ncbi.nlm.nih.gov/nuccore/ OR871751]

Research involving human participants, their data, or biological material

Policy information about studies with human participants or human data. See also policy information about sex, gender (identity/presentation), and sexual orientation and race, ethnicity and racism.

Reporting on sex and gender	NA
Reporting on race, ethnicity, or other socially relevant groupings	NA
Population characteristics	NA
Recruitment	NA
Ethics oversight	NA

Note that full information on the approval of the study protocol must also be provided in the manuscript.

Field-specific reporting

Please select the one below that is the best fit for your research. If you are not sure, read the appropriate sections before making your selection.

☐ Life sciences ☐ Behavioural & social sciences ☒ Ecological, evolutionary & environmental sciences

For a reference copy of the document with all sections, see nature.com/documents/nr-reporting-summary-flat.pdf

Ecological, evolutionary & environmental sciences study design

All studies must disclose on these points even when the disclosure is negative.

Study description	We collected 789 samples from 23 species from 8 study sites and 3 wildlife rehabilitation centers. We calculated SARS-CoV-2 RNA prevalence (# positives/# samples) for each species and reported 95% confidence intervals using the Agresti-Coull method to account for small sample sizes. We calculated SARS-CoV-2 seroprevalence and reported 95% confidence intervals using the Agresti-Coull methodfrom 67 wildlife samples prior to SARS-CoV-2 arrival and collected 49 samples during our study in 2022 from 6 species of wildlife. We evaluated the relationship between seroprevalence and surface imperviousness and human presence utilizing two logistic generalized linear mixed models with species as a random intercept.
Research sample	We collected samples from mammal species that were captured at eight field sites or whom were patients at local wildlife rehabilitation centers. We collected samples from 23 species (789 individual samples) whom are regularly observed in Virginia. Species included Blarina brevicauda (3), Didelphis virgininiana (34), Eptesicus fuscus (1), Lasiurus borealis (8), Marmota monax (1), Mephitus mephitus (7), Peromyscus leucopus (16), Peromyscus maniculatus (172), Procyon lotor (25), Sciurus carolinensis (19), Tamias striatus (8), Ursus americanus (2), Vulpes vulpes (1). The following species were being treated at veterinary clinics and samples were obtained from them for this study: Castor canadensis (2), Didelphis virgininiana (106), Eptesicus fuscus (6), Lasiurus borealis (4), Lynx rufus (3), Marmota monax (30), Mephitus mephitus (18), Microtus pennsylvanicus (1), Mus musculus (1), Mustela vison (1), Odocoileus virginianus (20), Peromyscus leucopus (3), Peromyscus sp. (2), Procyon lotor (59), Rattus rattus (2), Sciurus carolinensis (86), Sylvilagus floridanus (118), Tamias striatus (4), Urocyon cinereoargenteus (2), Ursus americanus (1), Vulpes vulpes (16)
Sampling strategy	No statistics were performed to determine the sample size. We attempted to collect as many samples as possible because we expected prevalence rates to be low.
Data collection	We collected nasopharyngeal or oropharyngeal samples from wildlife across 43 counties in Virginia and Washington D.C., U.S.A. We collected samples from three wildlife rehabilitation centers in Boyce, Roanoke, and Waynesboro, VA. In addition, we actively captured wildlife from eight sites between 2022 and 2023 that spanned a rural to urban gradient in Giles, Montgomery, Roanoke, and Wythe counties. All samples collected at the wildlife rehabilitation samples were collected by trained personnel employed at those centers. All samples collected at the 8 field sites were collected by Amanda Goldberg, Joseph Hoyt, Kate Langwig, Chris Kailing, Macy Kailing, and field technicians trained and overseen by one of the coauthors listed. Data was originally collected using pen and

	paper and then entered into an Excel database.
Timing and spatial scale	We trapped each of six sites for a 2-4 day session between 9-May and 1-July 2022 (two sites were trapped for two sessions with at least 27 days between them). Additionally, we trapped four sites for a 4-day session between 27-June and 11-September 2023. All field sites were located within a 1 hour drive of Blacksburg, VA and spanned an urbanization gradient. All samples from veterinary clinics were obtained between 1-July-2022 and 14-Sept 2023 with sample sizes being highest in the late spring/summer when they receive the largest number of individuals for care.
Data exclusions	NA
Reproducibility	All serological samples were processed in triplicate technical replicates. All attempts to repeat experimental work were successful.
Randomization	We tried to sample at sites located along an urbanization gradient. We were restricted in where we could sample by access but tried our best to find sites that varied in both humans who lived in the area and human visitation numbers. All other grouping was done by species so no randomization is required.
Blinding	All individual identification numbers are not provided to the lab when conducting RT-qPCR. However, they must know the species in order to apply the correct primers.

Did the study involve field work? ☒ Yes ☐ No

Field work, collection and transport

Field conditions	No field work was conducted in heavy rainfall or below freezing temperatures for animal safety concerns. During hotter months, we finished field work early in the morning to avoid heat stress to animals and conducted all animal processing in the shade. All animals were processed in the field where they were captured and released at the exact location of capture. Samples were collected from across Virginia and Washington D.C.
Location	We had two urban sites located in Blacksburg, VA (BB) and Roanoke, VA (RK). All homes were located in residential neighborhoods composed of single family or multiunit houses. Our locations in Roanoke were all on public land located in three small urban parks along the Roanoke River Greenway or on Mill Mountain Park. The small parks are located along a major bike/walking paved trail that runs along a river through the center of Roanoke. These parks were all located in mixed-use areas that included nearby houses, apartment buildings, hospitals, and businesses. Preston Forest (PF) is an intermediary site between urban and rural. Houses are spaced further apart (~5 acre lots) and hence the human population is at a lower density. Furthermore, there are no sidewalks so foot traffic is reduced. We trapped in the backyards of two homes that are mainly dominated by woody vegetation and leaf debris. New River Trails State Park (Foster Falls; NRT) is a section of a larger State Park located along the New River. Foster Falls has a campground and is a popular area for outdoor recreation. Brush Mountain (BM) is located within a complex of private, non-profit owned lands that are open to the public for hiking and biking. The area we trapped was not yet open to the public as it was in the process of being prepared (building a parking area, grooming trails, etc). Mountain Lake Biological Station site (ML) is a research field station with a rotating group of people using the facilities, including classrooms and housing. We trapped away from the housing complex in the surrounding forest. There was a hiking trail located adjacent to our sites and a commercial lodge about two miles down the road. However, the lands owned by the research station, where we trapped, were off-limits to public use. Pandapas Pond (PP) is located in Montgomery county and is a high use area for outdoor recreation including trail running, hiking, mountain biking, and fishing. We placed traps along a forest service road. The area is dominated by trees and leaf debris on the ground. Caldwell fields (CF) is located also in Montgomery county. The area is lightly used for general outdoor recreation including hunting.
Access & import/export	We had permits to access all field sites and were in regular contact (phone/email) with all parties involved. We obtained verbal/email access to land operated by New River Trails and private properties (backyards). We obtained the following permits for access to additional field sites: United State Forest Service (SF299 and nominal use), Virginia State Parks permit, Mountain Lake Biological Station permit, and Roanoke Parks and Recreation. Additionally we obtained a scientific collection permit from the State of Virginia to conduct our research at each of these locations
Disturbance	Minimal disturbance as we only drove on approved roads and walked to trap locations. Traps were only left at a site for 4 nights so minimal damage to local flora occurred.

Reporting for specific materials, systems and methods

We require information from authors about some types of materials, experimental systems and methods used in many studies. Here, indicate whether each material, system or method listed is relevant to your study. If you are not sure if a list item applies to your research, read the appropriate section before selecting a response.

Materials & experimental systems

n/a	Involved in the study	
☐	☒	Antibodies
☒	☐	Eukaryotic cell lines
☒	☐	Palaeontology and archaeology
☐	☒	Animals and other organisms
☒	☐	Clinical data
☒	☐	Dual use research of concern
☒	☐	Plants

Methods

n/a	Involved in the study	
☒	☐	ChIP-seq
☒	☐	Flow cytometry
☒	☐	MRI-based neuroimaging

Antibodies

Antibodies used

SARS-CoV-2 Delta virus strain USA-GNL-1205/2021 (a generous gift from the World Reference Center for Emerging Viruses and Arboviruses).

Validation

(ASK JAMES)

Animals and other research organisms

Policy information about studies involving animals; ARRIVE guidelines recommended for reporting animal research, and Sex and Gender in Research

Laboratory animals

No Laboratory animals were used in this study.

Wild animals

No animals were housed. All animals were captured in the field, processed at the field site, and immediately released at the location they were captured. We captured in total the following species (sample size in parentheses): Blarina brevicauda (3), Didelphis virgininiana (34), Eptesicus fuscus (1), Lasiurus borealis (8), Marmota monax (1), Mephitus mephitus (7), Peromyscus leucopus (16), Peromyscus maniculatus (172), Procyon lotor (25), Sciurus carolinensis (19), Tamias striatus (8), Ursus americanus (2), Vulpes vulpes (1). The following species were being treated at veterinary clinics and samples were obtained from them for this study: Castor canadensis (2), Didelphis virgininiana (106), Eptesicus fuscus (6), Lasiurus borealis (4), Lynx rufus (3), Marmota monax (30), Mephitus mephitus (18), Microtus pennsylvanicus (1), Mus musculus (1), Mustela vison (1), Odocoileus virginianus (20), Peromyscus leucopus (3), Peromyscus sp. (2), Procyon lotor (59), Rattus rattus (2), Sciurus carolinensis (86), Sylvilagus floridanus (118), Tamias striatus (4), Urocyon cinereoargenteus (2), Ursus americanus (1), Vulpes vulpes (16)

Reporting on sex

We did collect data for each sex. Sex was determined from external genitalia. Both sexes were combined for analyses.

Field-collected samples

We collected nasopharyngeal or oropharyngeal samples from each capture and serum from a subset of captures. All samples were collected when we processed animals. All samples were collected as quickly as possibly following approved protocols while individuals were closely monitored for any signs of distress.

Ethics oversight

Protocols were approved under Virginia Tech IACUC protocol #22-061.

Note that full information on the approval of the study protocol must also be provided in the manuscript.

Plants

Seed stocks

NA

Novel plant genotypes

NA

Authentication

NA
